# Prenatal Hyperhomocysteinemia Leads to Synaptic Dysfunction and Structural Alterations in the CA1 Hippocampus of Rats

**DOI:** 10.3390/biom15020305

**Published:** 2025-02-19

**Authors:** Tatyana Y. Postnikova, Alexandra V. Griflyuk, Natalia L. Tumanova, Nadezhda M. Dubrovskaya, Anastasia V. Mikhel, Dmitriy S. Vasilev, Aleksey V. Zaitsev

**Affiliations:** Sechenov Institute of Evolutionary Physiology and Biochemistry, Russian Academy of Sciences, 194223 St. Petersburg, Russia; tapost2@mail.ru (T.Y.P.); griflyuk.al@mail.ru (A.V.G.); natalia.tumanova@iephb.ru (N.L.T.); ndub@mail.ru (N.M.D.); anastasia.michel39@gmail.com (A.V.M.); aleksey_zaitsev@mail.ru (A.V.Z.)

**Keywords:** homocysteine, maximal electroshock threshold test, hippocampus, CA1, synaptic vesicles, field postsynaptic potentials, excitatory neurotransmission, prenatal development

## Abstract

Prenatal hyperhomocysteinemia (HCY) is associated with neurodevelopmental deficits, yet its long-term impact on hippocampal synaptic function remains poorly understood. This study examines the effects of moderate maternal HCY on excitatory synaptic transmission in the CA1 region of the dorsal hippocampus in rat offspring at juvenile (P21) and adult (P90) stages. Using field postsynaptic potential (fPSP) recordings, electron microscopy, and Western blot analysis, we observed a significant age-dependent decline in the efficiency of excitatory synaptic transmission in HCY-exposed rats. Electron microscopy revealed structural alterations, including synaptic vesicle agglutination in the stratum radiatum, suggesting impaired neurotransmitter release. Additionally, a significant reduction in pyramidal neuron density was observed in the CA1 region, although seizure susceptibility remained unchanged. Western blot analysis showed altered expression of Synapsin I, indicating presynaptic dysfunction. These findings suggest that moderate prenatal HCY leads to persistent deficits in synaptic transmission and structural integrity, potentially contributing to cognitive impairments in adulthood. Our results highlight the importance of maternal homocysteine levels in shaping hippocampal function and could offer insights into neurodevelopmental disorders associated with metabolic disturbances.

## 1. Introduction

Hyperhomocysteinemia (HCY) is a pathological condition characterized by elevated levels of homocysteine, a toxic non-proteinogenic amino acid, in body tissues. This condition can arise from genetic defects in enzymes involved in amino acid metabolism or from deficiencies in essential coenzymes, such as folic acid and B vitamins, due to dietary imbalances [1]. Homocysteine plays a central role in methionine metabolism, with its levels tightly regulated by two major pathways: remethylation and transsulfuration. Remethylation occurs through two distinct pathways. The folate-dependent pathway relies on the presence of folic acid (B9) and vitamin B12, as N-5-methyltetrahydrofolate donates a methyl group in a reaction catalyzed by methionine synthase. This pathway is critical for maintaining intracellular homocysteine balance and its circulation in blood plasma [2]. The folate-independent pathway, on the other hand, utilizes betaine as the methyl group donor. In the transsulfuration pathway, homocysteine is irreversibly converted to cysteine via reactions catalyzed by cystathionine β-synthase (CBS) and cystathionine γ-lyase (CSE), with vitamin B6 acting as a cofactor. This pathway contributes to glutathione synthesis, a vital antioxidant [3]. The dysregulation of these pathways, whether due to genetic mutations (e.g., in methylenetetrahydrofolate reductase (MTHFR) or CBS) or deficiencies in cofactors (e.g., vitamins B6, B12, or folate), can lead to homocysteine accumulation and the development of hyperhomocysteinemia [4,5].

Elevated homocysteine levels disrupt cellular metabolism and contribute to oxidative stress, endothelial dysfunction, and neurotoxicity, which are implicated in various pathological conditions, including cardiovascular diseases, neurodegenerative disorders, and developmental impairments [2,3,4]. For instance, homocysteine can induce thrombosis, degrade collagen and elastin fibers in blood vessel walls, and increase the risk of cardiovascular diseases [6]. Additionally, elevated homocysteine levels have been linked to microalbuminuria [7], aging, alcohol consumption [8], smoking [9], and neurodegenerative diseases [10].

Maternal HCY is a critical condition associated with elevated homocysteine levels in maternal and fetal tissues, often triggered by vitamin deficiencies (e.g., vitamin B12 and folate) [1,11] or genetic mutations such as MTHFR deficiency [12]. HCY is clinically defined as homocysteine levels exceeding 15 nmol/mL in the blood. Severe HCY (>100 nmol/mL) poses significant risks to fetal development, potentially leading to nervous system malformations or even fetal death [13]. Moderate (15–30 nmol/mL) and intermediate (30–100 nmol/mL) HCY are more common but still carry substantial risks.

Maternal HCY can impair placental function, disrupting the transport of oxygen, nutrients, and essential trophic factors to the fetus [14,15]. This condition is associated with fetal hypoxia and oxidative stress, which are key contributors to HCY pathogenesis [16,17,18,19]. Recent studies suggest that HCY may also affect neuronal activity and synaptic transmission in both peripheral and central neurons [20]. During critical periods of brain development, prenatal HCY can disrupt neuronal migration, leading to cortical dysplasia, a known factor in epileptogenesis [15,21]. For example, in a prenatal HCY model, the impaired radial migration of excitatory pyramidal neurons was observed in various cortical regions, while inhibitory interneurons remained unaffected [15]. Similar cortical dysplasia has been linked to long-term changes in neuronal excitability and intrinsic properties in the hippocampus and entorhinal cortex of young rats [22], consistent with findings in severe HCY models [23].

Severe HCY has been shown to increase brain tissue excitability, potentially leading to excitotoxicity [24]. In neonatal rats, severe maternal HCY increases seizure susceptibility [25] and enhances spontaneous neuronal activity in the hippocampus during the first postnatal week [23]. HCY also affects neonatal GABAergic signaling [26] and disrupts NMDA- and AMPA-receptor-mediated circuits [24,27]. While the effects of severe HCY on hippocampal excitability and synaptic transmission have been partially described [23,25,28], the impacts of moderate and intermediate HCY remain poorly understood.

Given the limited understanding of how prenatal HCY influences synaptic transmission in the cerebral cortex and hippocampus, this study aims to investigate the effects of moderate maternal HCY on synaptic transmission and plasticity in the dorsal hippocampus of rat offspring at juvenile (P21) and adult (P90) stages. The hippocampus, particularly the CA1 region, plays a critical role in learning, memory, and synaptic plasticity. While previous studies have shown that prenatal HCY exposure impairs synaptic plasticity and memory in offspring, the underlying mechanisms remain poorly understood.

To address this, we examined the long-term effects of moderate maternal HCY on excitatory synaptic transmission and synaptic ultrastructure in the dorsal hippocampus. Synaptic function was assessed using field postsynaptic potential (fPSP) recordings to measure synaptic transmission efficiency in the CA1 region. Structural changes in synaptic ultrastructure were analyzed using electron microscopy, while Western blot analysis was employed to evaluate the expression of synaptic proteins, including Synapsin I, a key regulator of presynaptic vesicle trafficking.

Our findings revealed that moderate prenatal HCY exposure led to a significant decline in synaptic transmission efficiency, particularly in adult rats. Electron microscopy showed the agglutination of synaptic vesicles in the stratum radiatum, indicating impaired neurotransmitter release. Additionally, we observed a reduction in pyramidal neuron density in the CA1 region, although seizure susceptibility remained unchanged. Western blot analysis demonstrated altered expression of Synapsin I, suggesting presynaptic dysfunction. These results provide new insights into the long-term consequences of prenatal HCY on hippocampal synaptic function and structure, highlighting potential mechanisms underlying cognitive impairments associated with this condition.

## 2. Materials and Methods

### 2.1. HCY Model

The maternal HCY model was performed on 5–6-month-old pregnant female Wistar rats. Female pregnancy was verified by the presence of sperm in vaginal smears after mating. The control group (n = 7) consisted of animals that were fed standard chow and given additional water per os. The HCY group (n = 7) consisted of female rats that received a 0.15% aqueous L-methionine (Sigma-Aldrich, Darmstadt, Germany) solution (600 mg/kg, which corresponded to 0.10–0.15 g per animal) daily from day 4 of pregnancy until delivery. Such a methionine load has previously been shown to increase homocysteine levels up to 15 nmol/mL in newborn blood and brain tissues, as was shown earlier in [29]. Offspring were collected for experiments at 3 weeks postnatally (P21) and at the adult stage at 3 months (P90). In the modeling of hyperhomocysteinemia, no significant differences in weight were observed between the control and experimental females prior to pregnancy or at the end of the second and third weeks of gestation. The number of offspring was also not different between the groups (12 ± 2). Hyperhomocysteinemia (HCY) is known to lead to reduced fetal and placental weight in the last trimester of pregnancy [30,31,32]. However, in the samples studied, there were no significant differences in the weight of P21 offspring rats between the HCY and control groups.

### 2.2. Brain Slice Preparation

A vibratome HM 650 V (Microm International, Walldorf, Germany) was used to cut horizontal brain slices (350 μm) as described previously [33]. The brains were rapidly extracted and immersed in a chilled, oxygenated (95% O_2_: 5% CO_2_) artificial cerebrospinal fluid (ACSF) containing the following: 126 mM NaCl, 24 mM NaHCO_3_, 10 mM dextrose, 2.5 mM KCl, 2 mM CaCl_2_, 1.25 mM NaH_2_PO_4_, and 1 mM MgSO_4_. Thereafter, the slices were subjected to an incubation at 35 °C for a duration of one hour. Following this, they were transferred to a recording chamber, where the flow rate of ACSF (30 °C) was kept at an average range of 6–7 mL/min.

### 2.3. Recordings of Field Postsynaptic Potential (fPSP)

We recorded fPSPs in CA1 using a glass microelectrode (<1.0 MΩ) filled with ACSF. We evoked synaptic responses by stimulating the Schaffer collaterals with a bipolar electrode made of insulated Nichrome wire placed in the *str. radiatum*. The dependence of the fPSP and fiber volleys (FVs) on stimulation intensity was determined using an A365 stimulus isolator (World Precision Instruments, Sarasota, FL, USA). Current intensity was increased in 25 μA increments from 25 to 200 μA.

Model 1800 Microelectrode AC Amplifier (A-M Systems, Carlsborg, WA, USA) was used to record the fPSPs. Digitization was performed with an ADC/DAC NI USB-6211 (National Instruments, Austin, TX, USA) using WinWCP v5 software (University of Strathclyde, Glasgow, UK). Clampfit 10.2 (Axon Instruments, Sunnyvale, CA, USA) was used for the offline analysis of the recordings. The maximum slope of the input–output (I/O) relationships (fPSP amplitude vs. FV amplitude) was calculated for each slice by fitting a sigmoidal Gompertz function, as described previously [33]:

(1) $$f=ae^{{-e}^{\left(-k\left(I-I_{i}\right)\right)}}$$

where *e* is Euler’s number (e = 2.71...); *a*, *k*, *I_i_* are function parameters. The maximum slope of the curve has been calculated as a*k/*e*.

The paired-pulse ratio (PPR) was measured by delivering paired pulses at 20 s intervals, with interstimulus intervals ranging from 10 to 500 milliseconds (ms). The PPR was subsequently calculated as the ratio of the second to the first fPSP amplitude.

### 2.4. Light Microscopy

As previously reported, we observed the death of pyramidal neurons and gliosis in the CA1 region of the hippocampus in young HCY rats [34]. In this study, we analyzed the viability of pyramidal neurons in adult (P90) HCY and control rats (n = 9 per group). Following transcardial perfusion with a 4% paraformaldehyde solution in 0.1 M PBS (pH 7.4), the brain tissue was further fixed in the same solution at 4 °C for two weeks. After removing the fixative, the tissue was immersed in a 20% sucrose solution in PBS (pH 7.4), frozen, and sectioned coronally using a Leica CM 1510S cryomicrotome (Leica Microsystems, Vetzlar, Germany). Sections of 15 µm thickness were obtained from the dorsal hippocampus, starting at the level of Bregma = −4.5 mm [35], with a 45 µm interval between sections.

The sections were Nissl-stained with cresyl violet and examined under an AF7000 microscope equipped with a DFC495 digital camera (Leica Microsystems, Vetzlar, Germany). This approach was used to detect morphological alterations in the CA1 and CA3 regions of the dorsal hippocampus. A total of 9 sections per animal were analyzed. Image segmentation and quantitative analysis were performed using VideoTest Morphology 4.0 software (VideoTest, St. Petersburg, Russia).

For counting neurons in the *str. pyramidale*, three criteria were applied: (1) localization within the *str. pyramidale* of the CA1 or CA3 region, (2) cell body size (>15 µm in diameter), and (3) pyramidal shape of the cell body (with the ratio of the longest to the shortest axis passing through the cell nucleus being at least 2). The mean number of pyramidal cells was counted within tissue areas (500 µm in the mediolateral axis and 1000 µm in the dorsoventral axis) encompassing the *str. pyramidale* and surrounding hippocampal layers in the CA1 and CA3 regions. The data are presented as the number of cells per 1 mm^2^.

### 2.5. Electron Microscopy

The CA1 region of the dorsal hippocampus was analyzed in three control and three HCY animals at P21 and 90 (P90). After perfusion with a solution of 1% glutaraldehyde and 1% paraformaldehyde in 0.1 M PBS (pH 7.4), the brain tissue was fixed in osmium tetroxide, contrasted with uranyl acetate, dehydrated, and embedded in Araldite. Sections were prepared using a Leica ultramicrotome (UC7 RT, Leica Microsystems, Vetzlar, Germany) and examined under a transmission electron microscope (FEI Tecnai Spirit V2, FEI, Hillsboro, OR, USA). For additional methodological details, please refer to [15].

In the quantitative analysis, three parameters of the neuropil were evaluated in the *str. radiatum* and *str. oriens* of the CA1 region: (1) the number of synaptic vesicles per synaptic terminal and varicose axonal extensions; (2) the mean area of synaptic terminals and varicose axonal extensions; and (3) the percentage of synaptic terminals with agglutinated synaptic vesicles relative to the total number of terminals and varicosities analyzed.

Image segmentation and quantitative analysis were performed using VideoTest Morphology 4.0 software (VideoTest, St. Petersburg, Russia). Approximately 24 CA1 hippocampal slices (with a mean area of about 1 mm^2^) were analyzed at P21 and P90, with 6 slices from 3 control rats and 6 slices from 3 HCY rats in each age group. From each animal, images of 2 slices containing either the *str. radiatum* or the *str. oriens* were obtained. A total of 124 patches containing synaptic terminals or varicose axonal extensions with clearly visible round synaptic vesicles (lacking dense cores) were analyzed. Terminals that did not meet these criteria or touched the image boundaries were excluded from the analysis.

Given the small sample size per animal (*n* = 3 per group) and the lack of significant differences between individual animals within each group (p = 0.14, two-tailed Kruskal–Wallis test), the data from individual rats were pooled into unified control (*n* = 56 terminals/varicosities) and HCY (*n* = 68 terminals/varicosities) groups. Differences between groups were assessed separately for the *str. radiatum* and *str. oriens*.

### 2.6. Western Blot

Tissue blocks from the dorsal hippocampus were obtained from P90 rats. The proteins were denatured, loaded, and separated by electrophoresis for 1–1.5 h at 120–150 V using a running buffer in a Bio-Rad electrophoresis system (Bio-Rad, Hercules, CA, USA). Following electrophoresis, the proteins were transferred onto PVDF membranes. The membranes were blocked and subsequently incubated overnight at 4 °C with primary antibodies: anti-Synapsin I antibody (1:1000 dilution; ab64581, Rabbit Polyclonal, Abcam, Cambrodge, UK) and anti-β-actin antibody (1:1000 dilution; ab8224, Mouse Monoclonal, Abcam, Cambrodge, UK). After washing with PBS-Tween, the membranes were incubated with HRP-conjugated secondary antibodies: Goat Anti-Mouse IgG (1:2000 dilution; ab205719, Abcam, Cambrodge, UK) and Goat Anti-Rabbit IgG (1:2000 dilution; ab205718) for 1 h. Protein bands were visualized using an ECL Substrate Kit (High Sensitivity; ab133406, Abcam, Cambrodge, UK).

### 2.7. Maximal Electroshock Seizure Threshold (MEST)

The susceptibility of rats to seizures was evaluated using the MEST (Maximal Electroshock Seizure Threshold) procedure at 2.5 months of age. Electrical stimulation was delivered through ear electrodes using an ECT Unit 57800 pulse generator (Ugo Basile, Gemonio, Italy). The stimulation current was calibrated on a logarithmic scale with 0.1 increments ranging from 12 to 100 mA, with a pulse frequency of 200 Hz, a pulse duration of 0.9 ms, and a stimulation duration of 0.8 s. For each animal, the minimum current required to elicit tonic extension of the hind limbs was determined.

This study began with a medium current of 40 mA. If tonic seizures with hind limb extension did not occur at this level, the current intensity was increased in the subsequent trial. Conversely, if tonic hind limb extension was observed at 40 mA, the current was decreased. Subsequent tests were conducted at intervals of 2–3 days.

### 2.8. Statistical Analysis

The data were analyzed using OriginPro, version 8.0 SR2 (OriginLab Corporation, Northampton, MA, USA), and Statistica, version 8.0 (StatSoft Inc., Tulsa, OK, USA). The normality of the data distribution was verified using the Kolmogorov–Smirnov test. Differences between the mean values of the two groups (control and HCY) were assessed for statistical significance using either an unpaired Student’s *t*-test or repeated measures ANOVA, as appropriate. For datasets with non-normal distributions, such as those obtained from electron microscopy and Western blot analyses, the non-parametric Mann–Whitney U-test was employed. All data are presented as the mean ± standard error of the mean (SEM). A *p*-value of less than 0.05 was considered statistically significant.

## 3. Results

### 3.1. Efficiency of Synaptic Neurotransmission at CA3-CA1 Is Reduced in HCY Rats

**Figure 1:**
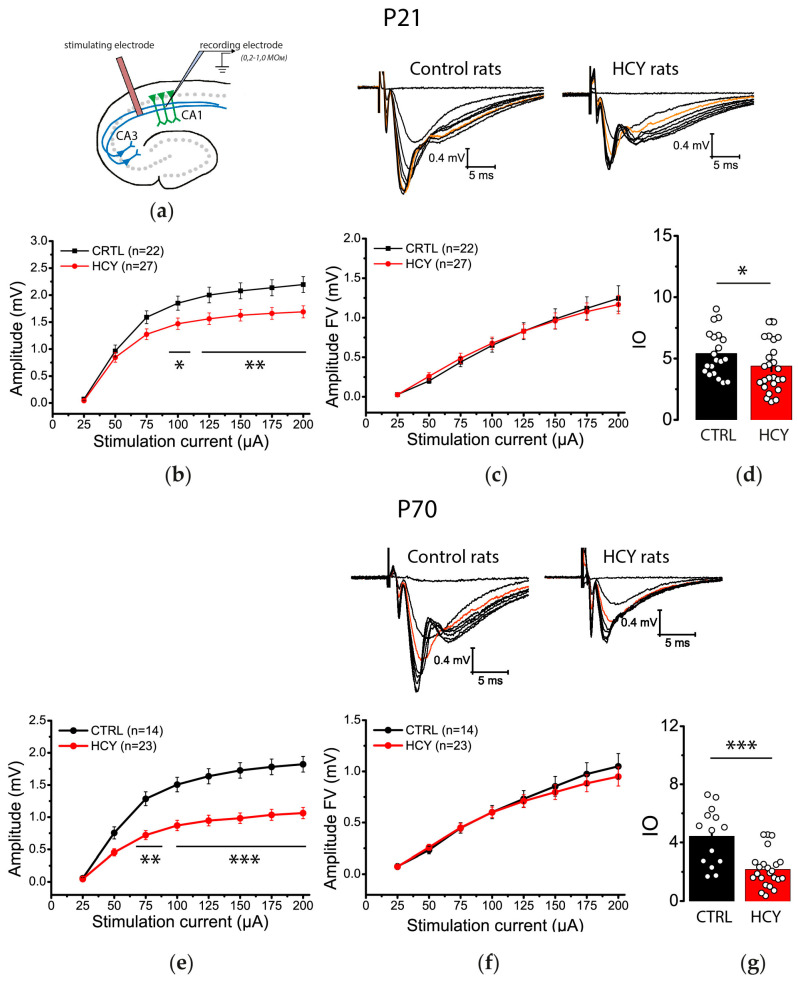
Hippocampal synaptic neurotransmission is reduced in HCY rats. (**a**) A schema showing the positions of electrodes in the hippocampus. The stimulation–response relationships for fPSP (**b**,**e**) and FV (**c**,**f**) amplitudes recorded from CA1 area and maximum I/O slopes (**d**,**g**) in the control (CTRL) and HCY rats (HCY) of different ages ((**b**–**d**)—P21, (**e**–**g**)—P70). Each point represents a single value. * *p* < 0.05, ** *p* < 0.01, *** *p* < 0.001—the difference between the control and HCY groups.

This study was designed to determine whether changes in synaptic transmission efficiency occur across CA3-CA1 pyramidal synapses in HCY rats at different ages (P21 and P70; Figure 1a). We measured the amplitudes of presynaptic FVs, which indicate the number of CA3 pyramidal cells firing action potentials, and fPSPs, which reflect the total postsynaptic response of CA1 neurons, using stimulation currents ranging from 25 to 200 μA.

At P21, the fPSP amplitude in HCY rats was significantly lower than in controls (repeated measures ANOVA, F_(7,329)_ = 6.13; *p* < 0.001, control: *n* = 22 slices; HCY rats: *n* = 27 slices; Figure 1b). In contrast, no significant differences in FV amplitudes were observed (F_(7,329)_ = 0.42; *p* = 0.89, Figure 1c). We further compared the input–output (I/O) relationships of fPSP and FV amplitudes between HCY and control rats. The maximum I/O slope, a measure of synaptic strength [36], was reduced by 29% in the HCY group (*t* = 2.40; *p* < 0.05; Figure 1d).

At P70, a significant decline in fPSP amplitude was observed in HCY rats compared to controls (repeated measures ANOVA, F_(7,245)_ = 19.88; *p* < 0.001; control: *n* = 14; HCY rats: *n* = 23; Figure 1e) across a broad range of stimulation currents (75–200 μA). However, no alterations in FV amplitudes were detected in rats (F_(7,259)_ = 0.67; *p* = 0.67; Figure 1f). Additionally, synaptic transmission was significantly attenuated at this age (*t* = 4.34; *p* < 0.001; Figure 1g), with a 51% reduction in the average I/O slope in HCY rats.

To assess age-related changes, we compared FV amplitudes between P21 and P70 within both the control and HCY groups. No significant differences in FV amplitudes were found between the two age groups in either the control (F_(1,34)_ = 0.29; *p* = 0.59; P21: *n* = 22; P70: *n* = 14) or the HCY rats (F_(1,50)_ = 0.61; *p* = 0.41; P21: *n* = 22; P70: *n* = 14). This suggests that the generation of action potentials in the axons of CA3 pyramidal neurons is not impaired in HCY rats and remains stable with age. Next, we conducted a similar analysis for fPSP amplitude and found that there were no age-related differences in the control group (F_(1,34)_ = 3,18; *p* = 0.08), whereas, in the HCY group, the differences were statistically significant (F_(1,50)_ = 19.3; *p* < 0.001).

In summary, these findings indicate that the efficiency of excitatory transmission at CA3-CA1 pyramidal neuron synapses is significantly diminished in HCY rats, with more pronounced deficits at P70 than at P21.

### 3.2. Short-Term Synaptic Plasticity (STP) of Hippocampal Neurons Is Altered in HCY Rats at P70

**Figure 2:**
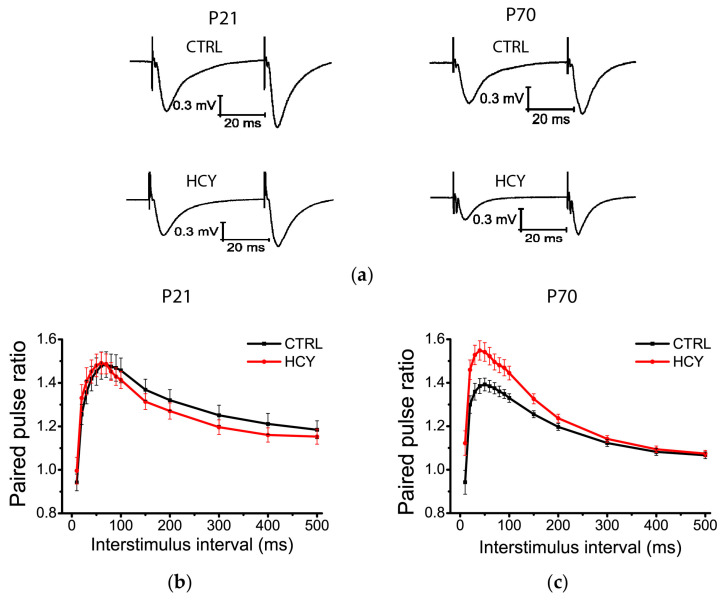
The paired-pulse ratio is altered in hippocampal slices from HCY rats at P70. (**a**) Representative examples of responses in control rats (CTRL) and HCY rats (HCY) with interstimulus intervals of 40 ms. (**b**,**c**) Plots of the PPR in hippocampal slices from rats at P21 (**b**) and P70 (**c**) at different interstimulus intervals. Each point represents the mean ± SEM.

Synaptic transmission efficiency can be influenced by disruptions in presynaptic mechanisms. Changes in STP properties can serve as an indicator of alterations in the probability of neurotransmitter release [37]. To investigate potential changes in STP, we employed paired-pulse stimulation and compared the amplitudes of fPSPs at intervals ranging from 30 to 500 ms in control and HCY rats (Figure 2).

Repeated measures ANOVA revealed no significant difference in the magnitude of short-term facilitation at P21 (F_(1,210)_ = 0.72; *p* = 0.41; control: *n* = 8; HCY rats: *n* = 9; Figure 2b). However, significant changes in the PPR were observed in the HCY rats at P70 (F_(14,476)_ = 4.74; *p* < 0.001; control: *n* = 13, HCY rats: *n* = 23; Figure 2c).

These findings suggest that the probability of glutamate release is altered in HCY rats at P70, indicating presynaptic dysfunction in this group.

### 3.3. Structural Properties of Synaptic Terminals in the Str. Radiatum of the CA1 Region Are Altered in Adult HCY Rats

**Figure 3:**
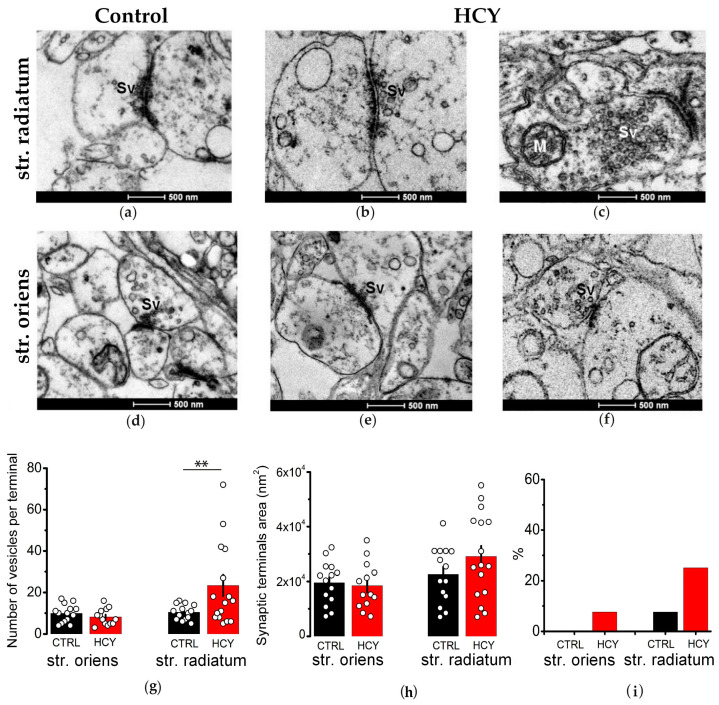
The ultrastructure of the synaptic terminals in the CA1 region of the control and HCY rats at P21. The figure shows electron microscopic images of synaptic terminals of axons in the *str. radiatum* (**a**–**c**) and *str. oriens* (**d**–**f**) of the CA1 area in the hippocampus of the control (**a**,**d**) and HCY (**b**,**c**,**e**,**f**) young rats. The scale bar is 500 nm. Sv—Synaptic vesicles; M—Mitochondria. Figures (**g**–**i**) show the number of synaptic vesicles per terminal (**g**), the mean area of the synaptic terminals (**h**), and the percentage of terminals with agglutinated synaptic vesicles out of the total number of synaptic terminals analyzed (**i**). Data are expressed as mean ± SEM. ** *p* < 0.01—the difference between the control and HCY groups, Mann–Whitney *U*-test.

We conducted an analysis of the ultrastructure of axon terminals in the *str. radiatum* and *str. oriens* of the CA1 region of the hippocampus in rats at P21, focusing on differences between the control and HCY groups. At this age, the majority of synaptic terminals in both groups appeared similar (Figure 3). However, in HCY rats, some synaptic terminals exhibited an increased number of vesicles. Quantitative analysis revealed that the mean number of vesicles in synaptic terminals was significantly higher in HCY rats compared to controls (Figure 3g), while the size of the axonal terminals did not differ between the groups (Figure 3h).

Additionally, rare cases of vesicle agglutination were observed in terminals located in the *str. oriens*, with more frequent occurrences in the *str. radiatum* (up to 25% of all terminals) in HCY rats. In contrast, the control group showed a lower agglutination rate of 7.6% in the *str. radiatum*, with no cases detected in the *str. oriens* (Figure 3i).

**Figure 4:**
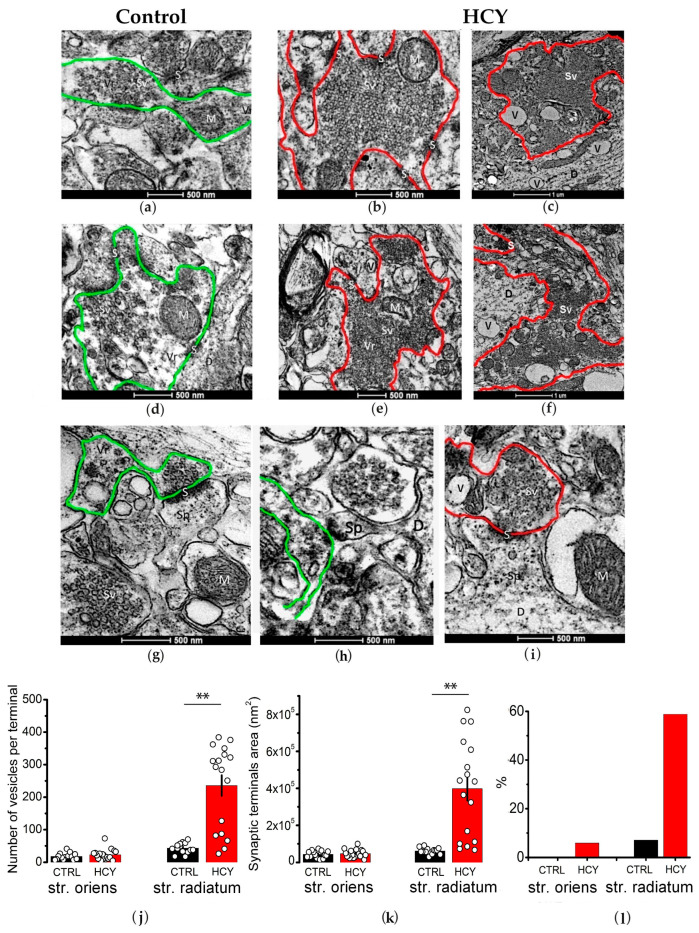
The ultrastructure of synaptic terminals in the CA1 region of the control (**a**,**d**,**g**,**h**) and HCY (**b**,**c**,**e**,**f**,**i**) rats at P90. The figure shows electron microscopic images of the synaptic terminals of axons. The accumulation and agglutination of synaptic vesicles in varicose extensions of axons in the HCY group are outlined in red. In the control group, axonal terminals are outlined in green. Sv—synaptic vesicles; M—mitochondria. Figures (**j**–**l**) show the number of synaptic vesicles per synaptic terminal (**j**), the mean area of synaptic terminals (**k**), and the percentage of terminals with agglutinated synaptic vesicles out of the total number of synaptic terminals analyzed (**l**). Data are expressed as mean ± SEM. ** *p* < 0.01—the difference between control and HCY groups, Mann–Whitney *U*-test.

In adult control rats, numerous varicose extensions of apical dendrites forming axospinous synapses were observed in the *str. radiatum*. Axonal terminals contained round synaptic vesicles with electron-transparent contents (Figure 4a,d,g,h), likely storing excitatory neurotransmitters such as glutamate or acetylcholine. In contrast, HCY animals exhibited pathological alterations in their axonal terminals (Figure 4b,c,e,f,i). These abnormalities included swelling and darkening of the axoplasm, as well as the accumulation and agglutination of synaptic vesicles. These changes were observed in terminals located on both the main apical dendrites and their thin outgrowths. Notably, synaptic vesicles accumulated more frequently in swollen axonal terminals compared to normal ones. In many cases, swollen axonal varicosities were entirely filled with synaptic vesicles (Figure 4b).

Our findings indicate that 65% of synaptic terminals in the *str. radiatum* of adult HCY rats exhibited swelling. The mean area of the terminals was significantly larger in the HCY group (398,230 nm^2^) compared to the control group (61,009 nm^2^; *p* < 0.001, *U* = 8, Mann–Whitney *U*-test; Figure 4k). Furthermore, the number of synaptic vesicles per terminal increased approximately fivefold in the HCY group (236 vesicles) compared to the control group (43 vesicles; *p* < 0.001, *U* = 20). In the *str. oriens*, no significant differences were observed between the two groups in terms of terminal size (*p* = 0.68, *U* = 108) or vesicle number (*p* = 0.41, *U* = 98).

In the HCY group, the agglutination of synaptic vesicles was frequently observed. These vesicles were round in shape and lacked electron-dense inclusions. Agglutinated vesicles were detected in 58.8% of the terminals/varicosities analyzed in the *str. radiatum*. In contrast, in control rats, agglutinated synaptic vesicles were observed in only 7.1% of the total terminals analyzed (Figure 4l). In the *str. oriens*, the agglutination of synaptic vesicles was observed in both groups but only occasionally.

Therefore, in the CA1 region of the dorsal hippocampus in HCY rats, there are pathological changes in axonal terminals and varicose extensions, as evidenced by their swelling and the agglutination of synaptic vesicles.

### 3.4. The Hippocampal Tissue of Adult HCY Rats Has Increased Levels of Synapsin 1

**Figure 5:**
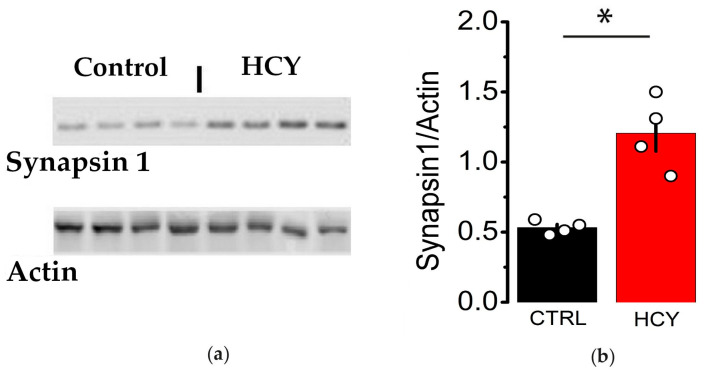
Hippocampal tissue from adult HCY rats has increased levels of Synapsin 1. (**a**) Representative bands of Synapsin 1 and β-actin in dorsal hippocampal tissue from adult control and HCY rats. (**b**) Bar graph showing results of densitometric analysis of Synapsin 1/actin ratio in control and HCY groups. * *p* < 0.05, *U*-test, *n* = 4 for each group. Original images of (**a**) can be found in Supplementary Materials.

Synapsin 1 is a phosphoprotein located on the cytoplasmic surface of synaptic vesicles [38]. Its removal from these vesicles mobilizes them from the reserve pool to the release-ready state, and it plays a role in the agglutination of synaptic vesicles, modulating neurotransmitter release [39]. In this study, we analyzed the content of this protein in the dorsal hippocampus tissue of adult HCY rats. Our analysis revealed a 50% decrease in Synapsin 1 content in the dorsal hippocampus tissue of adult HCY rats compared to the control group (*p* < 0.05, Mann–Whitney *U*-test, *n* = 4 for each group; Figure 5).

### 3.5. Maternal HCY Leads to a Decrease in the Number of Pyramidal Neurons in the CA1 Area of the Dorsal Hippocampus in Adult HCY Rats

**Figure 6:**
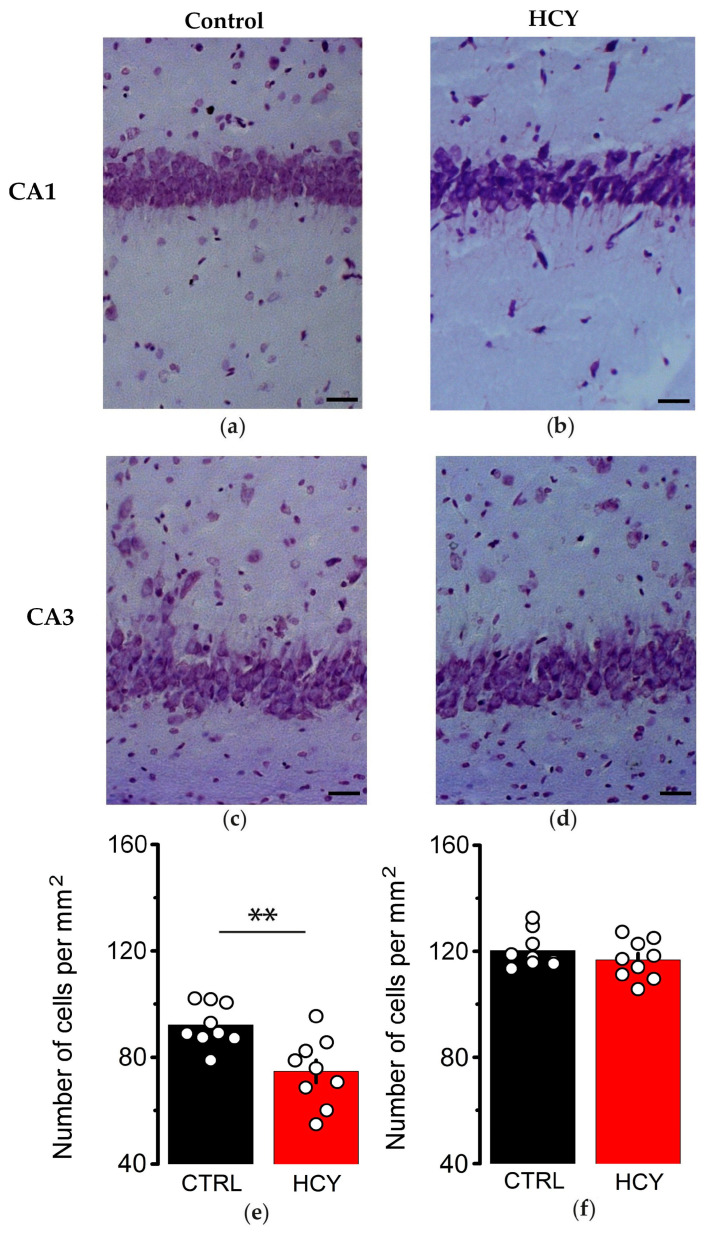
The loss of pyramidal neurons in the CA1 but not CA3 area of the dorsal hippocampus in adult HCY rats. (**a**–**d**)—Nissl staining of the CA1 (**a**,**b**) and CA3 (**c**,**d**) areas of the dorsal hippocampus in the control (**a**,**c**) and HCY (**b**,**d**) rats. The scale bar is 50 μm. The number of neurons in the CA1 (**e**) and CA3 (**f**) pyramidal layers of the hippocampus in adult control and HCY rats. ** *p* < 0.01—the difference between control and HCY groups, *t*-test, *n* = 9 for each group).

We previously detected pyramidal neuron loss and gliosis in the hippocampus of young HCY rats [34]. In this study, we analyzed whether the number of pyramidal neurons in adult HCY rats was reduced in the CA1 and CA3 hippocampal areas. Using Nissl staining, we compared the number of neurons in the pyramidal layer in adult control and HCY rats. Our findings revealed a significant decrease in the number of pyramidal neurons in the CA1 region of adult HCY animals compared to the control group (*t* = 3.48, *p* < 0.01, Figure 6). However, no differences in cell number were found in the CA3 region of the hippocampus (*t* = 1.05, *p* = 0.31). No obvious morphological changes in pyramidal neurons were observed, suggesting that cell death occurred at earlier stages of postnatal development.

### 3.6. No Change in Maximum Electroshock Seizure Threshold in HCY Rats

**Figure 7:**
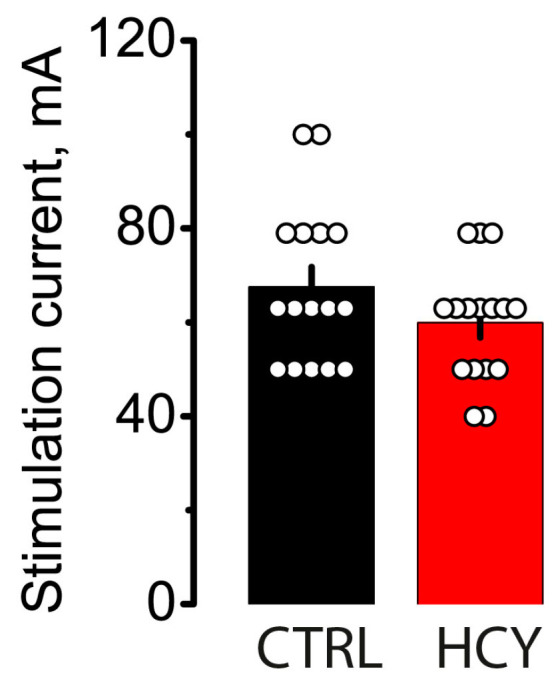
The seizure threshold is unchanged in the HCY rats at P70. Graphs illustrating the results of the MEST test in the control (CTRL) and experimental (HCY) animals. Circles show individual values for each rat.

Recently, rats with prenatal HCY were shown to be more susceptible to flurothyl-induced seizures in vivo and to 4-aminopyridine-induced epileptiform activity in the hippocampus in vitro [25]. In the present study, the MEST test was performed on HCY rats (*n* = 16 rats) and control animals (*n* = 16 rats) at P70 to investigate seizure susceptibility. HCY rats showed no change in the threshold for tonic hind limb extension (*U* = 98.5, *p* = 0.27, Figure 7).

## 4. Discussion

In the present study, we investigated the long-term consequences of moderate maternal HCY exposure on synaptic transmission in the dorsal hippocampus of juvenile (3-week-old) and mature (3-month-old) rats. Recordings of fPSPs in the CA1 region revealed that HCY rats exhibited impaired synaptic neurotransmission efficiency, with these impairments worsening with age. Furthermore, the PPR of fPSPs was increased, indicating presynaptic impairment of synaptic transmission. Electron microscopy revealed agglutination of synaptic vesicles containing glutamate and/or acetylcholine in the *str. radiatum* of the CA1 region of adult HCY rats, suggesting impaired exocytosis of excitatory mediators at CA3-CA1 synapses.

Several factors may be involved in alterations in the excitability of neuronal networks, including changes in synaptic transmission and its modulation. Basic synaptic transmission is maintained by the proper organization of synaptic vesicle pools. The disruption of neurotransmission may result from prolonged neuronal activity whereby the continuous exocytosis of neurotransmitters predominates over mechanisms that replenish neurotransmitter availability. Such phenomena, for example, may occur during epileptic seizures, causing significant changes in the dynamics of vesicle redistribution at the synapse. Studies of rat central nervous system synapses after seizures have revealed two opposite changes: an immediate abrupt depletion of synaptic vesicles [40], similar to what is observed after other intense stimulations [41,42]. Additionally, there is a consistent increase in the number of vesicles near the active zone [43,44]. This movement of vesicles toward the synaptic cleft has been shown to promote epileptic focus activity by increasing neurotransmitter supply [45]. On the other hand, synaptic exhaustion during epileptic activity may serve as a protective mechanism against excessive nervous system excitability. However, enhancing excitatory synapses may adversely impact seizure prognosis.

In our model of moderate prenatal HCY, we observed the accumulation of agglutinated excitatory synaptic vesicles in synaptic terminals in the *stratum radiatum* of the hippocampus. Concurrently, baseline neurotransmission was reduced to a significant extent. Our hypothesis is that the decrease in neurotransmission, along with the increase in the reserve pool of synaptic vesicles, is related to a decrease in the release of glutamate in the hippocampal synapses. To test this hypothesis, we studied the features of short-term synaptic plasticity (STP) in hippocampal CA3-CA1 synapses, since this form of synaptic plasticity is associated with molecular changes in presynaptic terminals [37,46].

The majority of forms of STP are initiated by brief periods of activity that result in transient calcium accumulation within presynaptic nerve terminals. When a cell is exposed to two stimuli with a brief interstimulus interval, the response to the second stimulus may exhibit enhancement (facilitation) or suppression (depression) in comparison to the response to the initial stimulus [37]. The depression of paired stimuli is typically observed at short interstimulus intervals (less than 20 ms) and is likely attributable to the inactivation of potential-dependent channels or the temporary depletion of the reserve of vesicles located in the presynaptic terminals that are ready to be released. Conversely, many synapses demonstrate the facilitation of paired impulses at longer interstimulus intervals (20–500 ms) [46]. This facilitation is attributed to the calcium remaining after the initial action potential, which contributes to additional release during the subsequent stimulation [46].

The variation in the response to the secondary stimulus is directly associated with the probability of neurotransmitter release at the primary stimulus. Mediator release is the result of a complex series of processes that commence with the arrival of an action potential, which depolarizes the presynaptic terminal. This, in turn, leads to the entry of calcium ions through potential-dependent channels and the subsequent triggering of the molecular mechanisms that facilitate vesicle fusion with the presynaptic membrane. The release of mediators is contingent on three primary factors: the number of vesicles that are immediately available for release, the calcium concentration in the presynaptic terminal, and the efficient operation of all molecular mechanisms that ensure calcium signal reception and vesicle fusion [47]. Accordingly, the probability of mediator release depends on each of these factors. The STP data indicate that the probability of mediator release in hippocampal synapses is significantly reduced in animals in the moderate HCY model. These changes suggest potential disruptions in neurotransmitter release mechanisms, which could indirectly implicate calcium signaling. However, further studies would be required to directly examine calcium dynamics in this context.

We have previously demonstrated neurodegenerative changes in the brain tissue of young HCY rats [29,34]. Moderate HCY has been shown to induce neuronal death in excitatory pyramidal neurons within the brain cortex [29] and dorsal hippocampus [34] of young rats, accompanied by the onset of neuroinflammation during the initial postnatal month. The neuroinflammatory processes were most pronounced in the CA1 area of the dorsal hippocampus, which plays a critical role in the organization of complex behavior in rats [34,48]. On the other hand, caspase-associated apoptosis was shown to be a minor mechanism of neuronal death in the HCY model [34,48].

In the present study, we observed a decline in the number of pyramidal neurons in the CA1 area of adult HCY rats, but not in the CA3 area. This decline could be indicative of neuronal death, as evidenced by observations at P20–30, despite the absence of pathological changes in the pyramidal neurons of adult HCY rats. Our findings are consistent with those reported in another study [49], which also demonstrated that HCY results in neuronal death. The mechanisms of neuronal death in the brain tissue of HCY offspring remain poorly understood. While hypoxic mechanisms have been shown to play a significant role during the period of elevated homocysteine levels [17], both apoptotic [50] and inflammatory [48] mechanisms might play a more important role in offspring pups.

The data obtained suggest impaired exocytosis of excitatory mediators at the CA3-CA1 synapses, indicating that excitotoxic mechanisms might play a relatively minor role in adult rats after moderate HCY. However, in severe HCY models [23,24,25,28], excitotoxicity has been suggested to be a crucial mechanism of neurodegeneration.

Earlier we described the impaired function of synaptic transmission and the reduction in the pool of labile mushroom-shaped dendritic spines with spine apparatus involved in long-term potentiation in both young and adult HCY rats [51]. The impairment of LTP was accompanied by a decrease in memory functions described by many authors [51,52,53]. The decrease in the CA1-CA3 synaptic transmission efficiency described in the present study might also contribute to LTP impairment in HCY animals. There are data suggesting prenatal HCY might change the functions of different mediator systems. Besides the well-known effect of HCY on the glutamatergic system [23,24,25,28], HCY was shown to disturb the cholinergic system too [54].

On the other hand, the results of our studies suggest that moderate and intermediate maternal HCY did not lead to the increased excitability of neuronal circuitries in the brains of young rats. As the excitability and intrinsic neuronal properties of the hippocampus neurons were reported to be affected by rather severe HCY [23,24,25,28], it can be suggested that the effect of HCY on neural tissue excitability depends on the homocysteine level during early postnatal ontogenesis. In the model of severe HCY described in [23,25], females received daily methionine (7.7 g/kg body weight) with food, starting 3 weeks prior to pregnancy and 2 weeks after delivery [55]. The comparison of our data with the literature [23,24,25,28] suggests that the HCY effect on the neurons’ excitability in the hippocampus of offspring rats depends on the HCY severity.

The agglutination of the synaptic vesicles in the synaptic terminals and varicose extension of the axons in the *stratum radiatum* of HCY rats but not in the *stratum oriens* is an interesting ultrastructural feature of HCY-associated pathology in the hippocampus. It had never been reported previously in the literature on HCY. The size and round shape of the agglutinated vesicles make them distinct from both flattened oval GABAergic synaptic vesicles and catecholamine-containing vesicles characterized by the presence of an electron-dense conglomerate inside them and can be referred to as excitatory glutamatergic or cholinergic vesicles [56]. The accumulation of the agglutinated excitatory synaptic vesicles in the synaptic terminals and varicose extension of the axon in the *stratum radiatum* of HCY hippocampus might suggest the decreased release of excitatory mediators to prevent the overexcitation and excitotoxic death of the CA1 pyramidal neurons. It might be one of the adaptive mechanisms in mild but not severe HCY. A decrease in the excitatory mediator release in the stratum radiatum of CA1 might normalize the excitability of neuronal circuitries in mild HCY rats

The reserve vesicles are rather common for Shaffer collaterals. They are suggested to support neurotransmission indirectly, ensuring that soluble recycling proteins are delivered upon demand during synaptic activity [57]. The dramatic increase in the reserve pool of excitatory synaptic vesicles might be associated not only with the pathology of glutamate (or acetylcholine) exocytosis in the presynaptic terminal in the hippocampus of HCY rats but also with other processes not studied yet.

## 5. Conclusions

In this study, the long-term effects of moderate prenatal HCY on the synapse properties in the dorsal hippocampus in rats and neuronal network excitability were examined. The seizure threshold at maximum electroshock did not differ between the control and HCY groups, indicating that moderate HCY does not lead to brain hyperexcitability. Furthermore, adult rats exposed to moderate HCY exhibited a reduction in excitatory synaptic neurotransmission efficiency within the CA3-CA1 area. Furthermore, we observed the agglutination of synaptic vesicles containing glutamate and/or acetylcholine in the *str. radiatum* of the CA1 region of adult HCY rats, suggesting that exocytosis of excitatory mediators in CA3-CA1 synapses may be impaired. Consequently, our findings indicate that prenatal HCY leads to alterations in excitatory CA3-CA1 synaptic transmission, which may decrease neuronal network excitability and contribute to the observed memory impairments in adult rats.

## Data Availability

The data presented in this study are available on request from the corresponding author.

## Supplementary Materials

The following supporting information can be downloaded at: https://www.mdpi.com/article/10.3390/biom15020305/s1, Original images of Figure 5a can be found in supplementary materials.
