# Supplementary figures and images for: Prenatal Hyperhomocysteinemia Leads to Synaptic Dysfunction and Structural Alterations in the CA1 Hippocampus of Rats
Source: Biomolecules. 2025 Feb 19;15(2):305. doi: 10.3390/biom15020305 (PMC11852833; doi:10.3390/biom15020305)

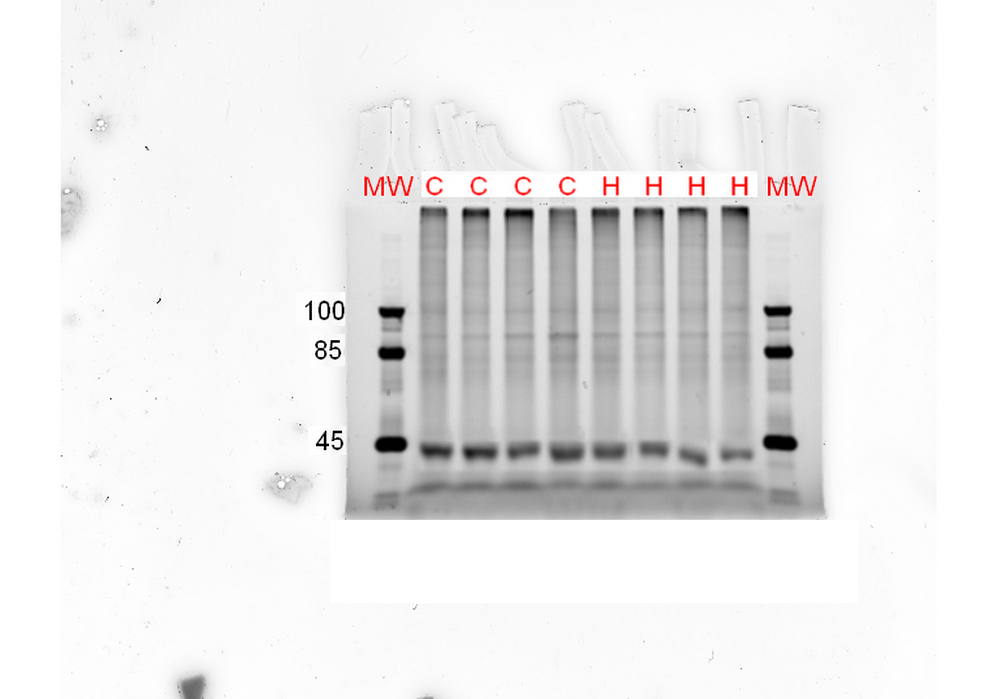

Supplement: Supplementary file 1 [file biomolecules-15-00305-s001.zip › actin.tif]

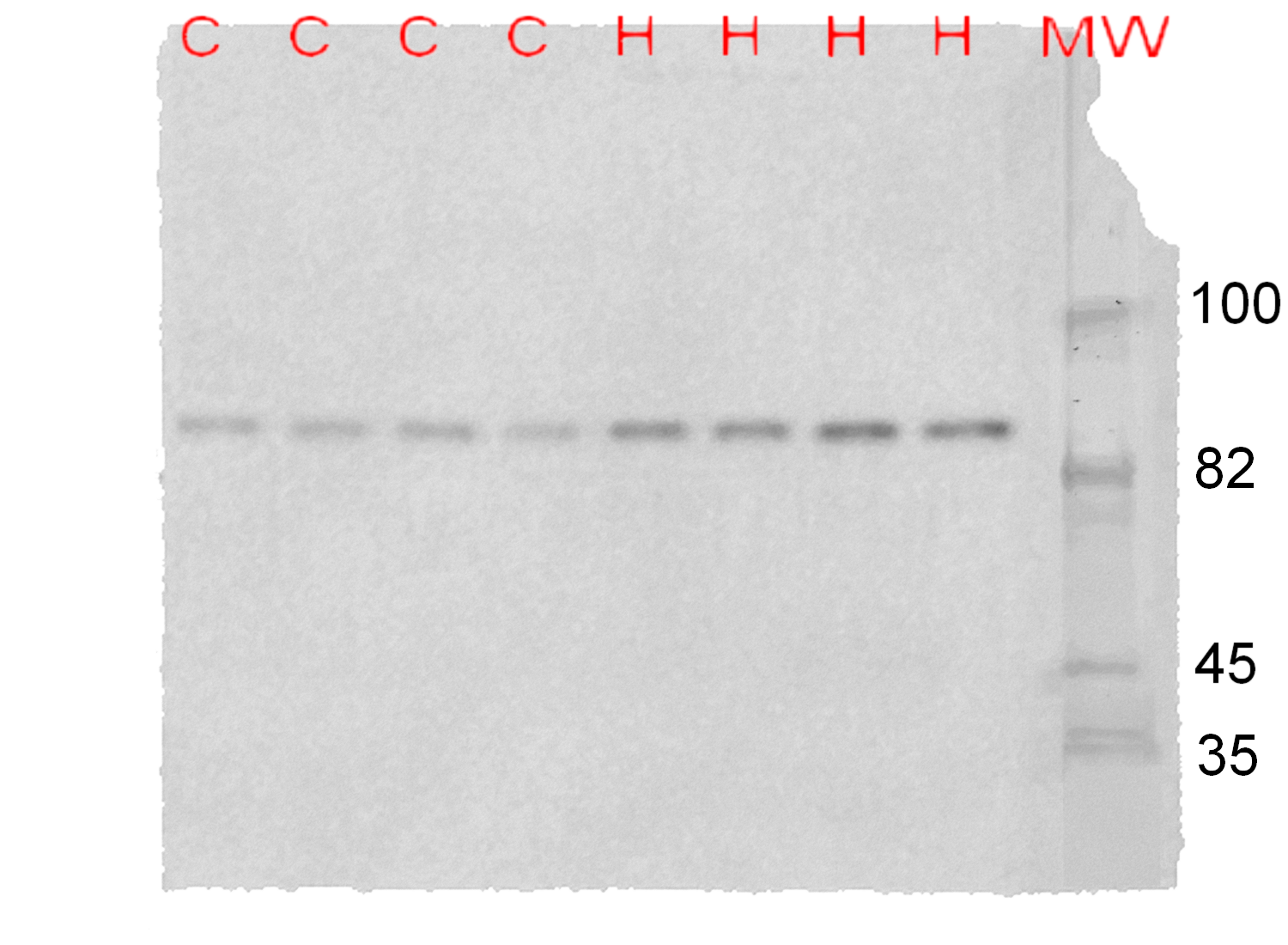

Supplement: Supplementary file 1 [file biomolecules-15-00305-s001.zip › Syn1-noncrop.tif]
